# Death of Dr. E. M. Nutting

**Published:** 1882-08

**Authors:** K. C. Moody


### DEATH OF DR. E. M. NUTTING.

But a few short months since the class of '82 went out from their Alma Mater, the University of Michigan, happy in the prospects for the future; yet, in that short time, man's last foe—Death—has entered our ranks and taken away our friend and classmate, Dr. E. M. Nutting, who passed away on the morning of July 3, at his home in East Randolph, N. Y. The members of the class will each feel a personal bereavement in this, our first loss by death.

MRS. K. C. MOODY,
Class Secretary.

